# Isolation and Characterization of Microalgae Isolates from Hydroponic Effluent Water: Metagenomics and Biotechnological Insights

**DOI:** 10.3390/microorganisms14030582

**Published:** 2026-03-04

**Authors:** Alexandros Ntzouvaras, Aikaterini Koletti, Maria Eleftheria Zografaki, Sofia Marka, Dimitrios Skliros, Gabriel Vasilakis, Ioannis Karavidas, Adonis Konstantinos Koukouvinis, Rodica C. Efrose, Chrysanthi Kalloniati, Ioannis Tzovenis, Emmanouil Flemetakis

**Affiliations:** 1 Laboratory of Environmental Biotechnology, Department of Biotechnology, Agricultural University of Athens, Iera Odos 75, 11855 Athens, Greece; alexntzouv@gmail.com (A.N.); kolettik@aua.gr (A.K.); mzografaki@aua.gr (M.E.Z.); smarka@aua.gr (S.M.); dskliros@aua.gr (D.S.); rodiefros@yahoo.com (R.C.E.); xkalloni@gmail.com (C.K.); 2 Laboratory of Food Microbiology and Biotechnology, Department of Food Science and Human Nutrition, Agricultural University of Athens, 11855 Athens, Greece; vasilakis.gavriil@gmail.com; 3 Laboratoty of Vegetable Crops, Department of Crop Science, Agricultural University of Athens, Iera Odos 75, 11855 Athens, Greece; karavidas@aua.gr; 4 Sector of Ecology & Systematics, Department of Biology, School of Science, National and Kapodistrian University of Athens, Panepistimiopolis Zografou, 15784 Athens, Greece; koukouvinis.ka@gmail.com; 5 Department of Chemistry, Physics and Environment, Faculty of Sciences and Environment, “Dunarea de Jos” University of Galati, 47 Domneasca, 800008 Galati, Romania; 6 Department of Marine Sciences, University of the Aegean, 81100 Mytilene, Greece; 7 Microphykos Aquaculture Systems & Services, Halandri, 15238 Athens, Greece; itzoveni@microphykos.com

**Keywords:** hydroponic effluent, microalgal isolates, metagenomics, characterization

## Abstract

Hydroponic systems are gaining prominence in sustainable agriculture, yet their nutrient-rich effluents remain an underexplored source of microbial biodiversity with potential biotechnological interest. In this study, shotgun metagenomic sequencing was employed to profile, with a high taxonomic resolution, the photosynthetic microbial community in hydroponic effluent before and after a natural algal bloom, revealing pronounced shifts in microbial composition. Notably, relative abundance increased sixfold for *Chlamydomonas reinhardtii* and tenfold for *Bigelowiella natans*. Four dominant microalgal strains (PR1–PR4) were subsequently isolated and characterized through integrative morphological and molecular taxonomy, with phylogenetic analyses based on four genetic markers (18S rRNA, ITS, *rbcL* and *tufA*) confirming that each isolate represents a distinct lineage within Chlorophyceae families, including *Chlorella* sp., *Chlamydomonas* sp., and *Scenedesmus* sp. Growth kinetics under three temperature regimes, typical of Greek environmental conditions from spring to autumn (15 °C, 23 °C, 32 °C), demonstrated broad ecological plasticity and rapid biomass production, highlighting strains with strong adaptive resilience. Biochemical profiling of the isolates revealed significant inter-strain differences in primary and secondary metabolite content, including proteins (up to 43% DW), lipids (up to 31% DW), carbohydrates (up to 44% DW), photosynthetic pigments, phenolics, flavonoids, and antioxidant activity. The observed metabolic diversity of autochthonous microalgal strains from hydroponic environments, combined with their high growth rates, underscores their potential for applications in bioremediation, bioenergy, and the development of value-added products within a circular bioeconomy framework.

## 1. Introduction

Microalgae are microscopic organisms belonging to various taxonomic groups found in diverse aquatic environments. They play a crucial role in the global carbon cycle by acting as primary producers, converting sunlight into organic compounds through photosynthesis, and holding significant potential for biotechnological exploitation due to their wide range of applications [1]. They are pivotal in biofuel production, wastewater treatment and the food and nutraceutical sectors, providing a variety of valuable ingredients such as omega-3 fatty acids, proteins and pigments. Moreover, the extensive and largely unexplored biodiversity of microalgae further enhances their potential, as numerous species with unique metabolic capabilities remain uncharacterized. These underutilized species could provide novel solutions in fields like pharmaceutical development, bioplastics, and cosmetics [2].

As the global population is projected to reach 9.8 billion by 2050, intensive farming practices are escalating, further exacerbating environmental issues through the addition of excessive nutrients and biopesticides [3]. Despite a shift towards sustainable agriculture, especially organic farming, challenges persist in meeting global food demand while preserving the environment, as yields from organic agriculture fall short of conventional methods due to insufficient organic sources of nutrients [4].

A potential solution to address these challenges is hydroponics, a cost-effective technology offering higher yields and adaptability, while also mitigating environmental issues such as soil infertility and the impact of natural disasters [5]. Despite its advantages, a significant portion of the fertilizers used in hydroponic systems remains underutilized. The continuous discharge of untreated, nutrient-rich hydroponic effluent into water bodies, especially in developing countries, results in significant repercussions such as water pollution, eutrophication, and the degradation of water quality, ultimately causing adverse effects on freshwater ecosystems [6]. Environmental regulations, such as the EU Water Framework Directive (WFD; 2000/60/EC), require a reduction in nutrient discharge, making alternative treatment of the hydroponic effluents essential. Furthermore, the nutrient-rich nature of hydroponic effluents fosters the growth of various microorganisms, including microalgae, which can further degrade water quality. Therefore, controlling their growth is critical to maintaining the proper function of these systems [7]. An innovative approach aims to valorize nutrient-rich hydroponic effluents through microalgal cultivation, integrating wastewater remediation with biomass generation. Such an approach facilitates nutrient recovery and organic load reduction while producing bioactive, value-added compounds. Ultimately, this strategy supports safe effluent discharge and promotes water reuse, offering a sustainable solution for regions challenged by freshwater scarcity.

The ability of microalgae to adapt to specific growth conditions varies significantly, with autochthonous strains that are naturally acclimated to hydroponic effluents proving to be more competitive and efficient [8]. These native microalgae strains have a strong potential for bioremediation, as they can effectively reduce nutrient loads in effluents, thereby mitigating environmental impacts such as eutrophication in aquatic ecosystems caused by nutrient release [9]. Moreover, such strains could exhibit valuable traits, including high lipid or protein content, making them suitable for biofuel production or as supplements in agriculture and aquaculture. Additionally, utilizing autochthonous microalgal strains reduces the risk of ecological disruption and promotes ecological balance compared to using non-native commercial strains. The interactions between these native strains and microbial communities in hydroponic effluent could also lead to innovative approaches for improving water quality, such as controlling populations of harmful bacteria like phytopathogens [10]. Microalgae-based technologies are emerging as a promising and viable option for the circular bioeconomy, offering a potential solution to environmental challenges associated with hydroponic effluents, though they remain in the early stages of development [11].

However, a critical knowledge gap persists in dynamically and comprehensively profiling the microbial community within these engineered ecosystems. Traditional monitoring relies on microscopic identification or indirect proxies like chlorophyll a, which are labor-intensive, morphologically limited, or taxonomically blind [12]. This limitation hinders a predictive understanding of community assembly, stability, and function, especially during pivotal ecological transitions like algal blooms. Such blooms, whether occurring naturally or induced for bioremediation purposes, represent a drastic restructuring of the microbial network, with profound implications for nutrient cycling, system efficiency, and the quality of the resulting biomass [13,14]. Modern metagenomic approaches, particularly shotgun sequencing, offer a powerful lens to address this gap. This technique has elucidated how operational parameters (e.g., temperature, stagnation) shape microbial structure and function in engineered water systems, but its application to track community dynamics before and after induced algal blooms in hydroponic effluent contexts remains nascent [15].

Therefore, in the present study, we report an in-depth analysis of the changes in the photosynthetic microbial community structure of a hydroponic effluent upon natural bloom conditions, as revealed through metagenomic analysis. These changes, occurring after a natural algal bloom, reflect a substantial shift in the microbial community, including the enrichment of chlorophyte microalgae. Concurrently, dominant microalgal strains were isolated, and both morphological observations and molecular techniques were employed for their taxonomic identification. To further assess their biomass production and biotechnological potential, growth and kinetic experiments were conducted across various temperature regimes, and a basic characterization of the resultant microalgal biomass was performed, providing insights into their potential biotechnological applications.

## 2. Materials and Methods

### 2.1. Sampling and Nutrient Enrichment

Four effluent samples derived from industrial hydroponic crops located in Messolonghi, Greece (tomato plants, “PYK”- https://www.pyk.gr/, accessed on 20 February 2026) were collected after the end of the cultivation cycle without any other treatment, and selected physicochemical properties were measured (pH, temperature, dissolved O_2_ and salinity) using a Lovibond SensoDirect 150 system (Tintometer Ltd., Lovibond^®^ Water Testing, Amesbury, UK) and an Atago S/Mill-E (Atago Co., Ltd., Tokyo, Japan) (Supplementary Table S1). A plankton net with a mesh size of 25 μm was used to filter samples and obtain nano- and micro-planktonic species, simultaneously removing debris, predators and larger organisms. Each sample was transferred in 1.5 L transparent plastic containers, enriched with L1 culture medium and incubated under stable conditions of light (50 μmol photons m^−2^ s^−1^ and 12:12 h L/D cycle) and temperature (22 ± 1 °C) until algal blooms were achieved.

### 2.2. Metagenomics Analysis

#### 2.2.1. DNA Extraction

Water effluent samples were collected in triplicate into sterile plastic bottles and preserved at 4 °C for a short time. Three water samples of 2.5 L were collected before and after algal bloom. Following collection, samples were filtered through 25 mm diameter, 0.45 μm pore-size polycarbonate filters (Whatman plc, Maidstone, UK) using a vacuum filtration system. The filters were then transferred to cryovials and stored at −80 °C until further analysis. All glassware and collection bottles were cleaned before use with a 2% bleach solution, followed by thorough washing with ethanol and sterile water.

Total microbial community DNA was extracted using an SDS-based lysis buffer and phenol/chloroform-based extraction method [16], optimized for the volumes that were sampled. Briefly, extraction buffer contained 10 mM Tris (pH 7.2), 0.1 mM EDTA, 2% SDS, b-mercaptoethanol and proteinase k. Filters were homogenized with liquid nitrogen before adding the extraction buffer. Samples were then incubated for 2.5 h at 56 °C. The liquid phase containing nucleic acids was separated with centrifugation at 11× *g* for 15 min at 4 °C. Total nucleic acids were extracted twice for each sample (8 DNA samples from 4 effluent samples) by using an equal volume of phenol, chloroform, and isoamyl alcohol (25:24:1) before precipitation from the aquatic phase with isopropanol overnight at −20 °C. Total nucleic acids were washed with ethanol and resuspended in ddH_2_O. An RNAse step was followed at 37 °C for 1 h, and DNA was purified with a commercial DNA isolation kit (NucleoSpin gDNA Clean-up; Macherey-Nagel, Duren, Germany) according to the manufacturer’s protocol. DNA quality and approximate concentration were estimated using a NanoDrop spectrophotometer and a 0.7% agarose gel to monitor DNA fragmentation. For each effluent sample, both DNA isolations were pooled to increase the DNA yield.

#### 2.2.2. Shotgun Sequencing and Metagenomics Analysis

DNA samples were sequenced using a paired-end 150 bp shotgun sequencing approach. Approximately 5 μg of DNA were fragmented to an average size of about 400 bp for paired-end library construction. A paired-end library was constructed using the Nextera library preparation kit (Illumina Inc., San Diego, CA, USA). Paired-end sequencing was performed on an Illumina Novaseq 6000 sequencing platform (ADM Biopolis, Valencia, Spain) according to the manufacturer’s instructions. In total, four distinct DNA preparations and sequencings took place, two for each treatment before the algal bloom (BA) and after the natural algal bloom (AA), which were used for analyzing at least 20mil PE quality reads for each treatment for in-depth taxonomic analysis of photosynthetic microbes. In total, 93,856,404 reads were analyzed in the BA treatment, and 71,918,136 in the AA treatment. Alpha and beta diversity analyses took place for each sample separately. For taxonomy purposes, Kraken 2 [17] was utilized for exact-match database queries of k-mers using the NCBI Reference Sequence (RefSeq) database. Reads were filtered to remove Chordate phyllum, including *Homo sapiens* DNA. The Bray and Curtis dissimilarity index [18] and alpha diversity indexes were calculated using KrakenTools. Bracken was used for the taxonomic assignments made by Kraken 2 to estimate abundance at the species level [19].

#### 2.2.3. Mapping

The limitations of using the Kraken bioinformatics tool include its requirement for exact alignment of sequence reads with known organisms [17]. To further enhance the identification of well-known Mediterranean microalga species with relevant biotechnological value [20], we enriched our analysis with manual mapping against three green microalgae species. For the mapping process, the Geneious Mapper (version 10.2.6) was utilized with five iterations and a minimum mapping quality of 20, which corresponds to a 99% confidence score against the selected genomes. Three green microalgae were chosen as reference genomes. *Chlorella vulgaris* (NCBI Bioproject PRJNA495479), *Chlorella_variabilis* NC64A v1.0 (JGI Project Id 16663) and *Scenedesmus obliquus* (NCBI BioProject PRJNA887759).

To compare the number of reads assigned to photosynthetic microbes before and after the algal bloom with Kraken 2 and Geneious mapper, we normalized the data as per million reads. Both mapped and unmapped reads were included in the normalization process, utilizing only the high-quality reads.

### 2.3. Isolation of Microalgal Strains

The most abundant and dominant strains of the algal blooms were isolated using standard techniques, as follows: 0.1 mL of the parent culture was uniformly spread on L1 medium agar plates (0.9% *w*/*v*) under sterile conditions. The plates were incubated under the same conditions as the parent cultures until algal colonies were observed. A single-colony sample was extracted and spread across new agar plates. After streaking, the agar plate was incubated under stable conditions until microalgal colonies appeared.

For non-settling strains, the serial dilution technique was employed, with small volumes of the initial cultures transferred to test tubes under sterile conditions. Then, the same volume was transferred again from the first to the second tube and so on until the final tube was inoculated. The tubes were incubated under stable conditions until unialgal growth was observed. If not, the micropipette isolation was used. The sample containing cells of the target species was placed on a microscope slide, and sterile culture medium droplets were placed in a multiwell plate. Using capillary action, the desired cell was drawn up the micropipette tip and then gently discharged into a sterile droplet. The method was repeated several times to increase the possibility of obtaining a single viable cell. Finally, the multiwell plate was incubated under stable conditions until unialgal growth was observed, and then using upscaling techniques, monocultures of the isolated strains were developed.

### 2.4. Taxonomic Classification

For the comprehensive characterization of the isolated strains, both classical morphological and molecular taxonomy methods were employed. The strains were observed at different life stages using optical microscopy (Zeiss KF2 microscope, Jena, Germany) and the Nomarski interference contrast (NIC) technique. Genomic DNA was extracted from all isolates using the Genomic DNA from Soil kit (Macherey-Nagel, Düren, Germany), following the manufacturer’s protocol. For taxonomic classification, four molecular markers were targeted: the nuclear 18S rRNA gene, the internal transcribed spacer (ITS) region, and the chloroplast markers *rbcL* (ribulose-1,5-bisphosphate carboxylase/oxygenase) and *tufA* (elongation factor Tu). These fragments were amplified via PCR with a protocol optimized for microalgae-derived samples, as previously described (Supplementary Table S2). PCR products were purified using the NucleoSpin Gel and PCR Clean-up kit (Macherey-Nagel, Düren, Germany), following the manufacturer’s instructions. Direct sequencing of both strands of each PCR amplicon was performed using the same primers as in the PCR reactions (CEMIA, Larisa, Greece).

The nucleotide sequences generated in this study were processed using DNA Baser v5.08 software (Heracle BioSoft SRL, Lilienthal, Germany). Sequence homologies were identified through blastn (version 2.17.0) searches against the GenBank database. The related reference sequences retrieved from the GenBank database, along with the sequences generated in the present study, were selected for subsequent phylogenetic analysis. Sequence alignment was performed using the Clustal W algorithm implemented in MEGA X 10.2.2 software [21]. Phylogenetic trees for each DNA marker were generated separately using the Neighbor-Joining (NJ) method based on Kimura’s two-parameter model and the Maximum Likelihood (ML) method, applying the best-fit nucleotide substitution model for each molecular marker: the Kimura 2-parameter (K2) model [22] for the 18S rDNA and ITS regions and the General Time Reversible (GTR) model [23] for the *rbcL* and *tufA* genes. Bootstrap support values were calculated based on 1000 replicates. The DNA sequences obtained for the selected DNA markers were deposited in the NCBI database with the accession numbers PQ773481-PQ773488 and PQ800404-PQ800407.

### 2.5. Growth and Kinetics Evaluation

In a controlled culturing chamber ensuring constant conditions, 500 mL cultures were grown in L1 culture medium under artificial illumination provided by cool-daylight fluorescent lamps (865) at 200 μmol photons m^−2^ s^−1^. The photoperiod was set to 12:12 h (light/dark), with a temperature of 22 ± 1 °C and aeration at approximately 0.5 vvm (volume per minute) through a 0.2 μm filter. Cultures were maintained for 5 days, with inoculum harvested during the early stationary phase and subsequently scaled up to 2 L volumes under identical conditions. Henceforth, these culturing conditions will be referred to as ‘control conditions,’ as they correspond to the standard preservation conditions for these strains in the lab. During growth, abundance was assessed daily using a Neubauer Improved hemocytometer under a Zeiss KF2 microscope with lenses (×10, ×20) by Zeiss Planapo. To further study strains’ growth and kinetics, three different temperature regimes simulating environmental temperatures throughout the year were selected (15 °C, 23 °C and 32 °C). The rest of the cultivation conditions remained as previously described. All experiments were performed using triplicate cultures.

Maximum specific growth rate (d^−1^) was determined using the equation:

μ_max_ = ln(Nt/Nt_0_)/(t − t_0_)

t_0_: the start of the exponential phase (days); t: the end of the exponential phase (days); Nt: cell density at the end of the exponential phase (cells/mL); Nt_0_: cell density at the start of the exponential phase.

### 2.6. Biochemical Characterization of Microalgal Biomass

When the microalgal cultures reached the stationary phase, the biomass was harvested, lyophilized, and pulverized into a fine powder. To achieve a comprehensive biochemical characterization of the isolated microalgal biomass, various analytical protocols were applied to evaluate both primary and secondary metabolites. Among primary metabolites, total proteins were quantified using the Kjeldahl method (Foss Tecator Kjeltec 8200 Distillation Unit) with a nitrogen-to-protein conversion factor of 5.95 [24,25]. Total lipids were quantified by the phosphovanillin method [21], while total carbohydrates were measured using the phenol–sulfuric acid method [26].

To analyze the microalgae secondary metabolite composition, methanolic extracts were prepared from lyophilized biomass. Briefly, 20 mg of biomass was suspended in 0.5 mL of methanol and placed in an ultrasonic bath for 20 min at a 40 kHz frequency. The lysate was then centrifuged at 13,000 rpm, and the resulting supernatant was collected. The extraction procedure was repeated twice, and the supernatants were pooled for subsequent analysis. Throughout the extraction process, the temperature was maintained below 10 °C to prevent sample degradation.

The total antioxidant capacity was determined using the ABTS assay in a microplate and expressed as mM Trolox equivalents per gram of dry biomass. The total phenolic content of the microalgal species was determined using the Folin–Ciocalteu assay [27] and expressed as mg gallic acid equivalents per gram of dried biomass. Total flavonoids were quantified using the aluminum chloride colorimetric method [28] and expressed as mg catechin equivalents per gram of dried biomass.

For the determination of photosynthetic pigments, the same extraction process was applied to lyophilized biomass using 80% acetone in water as the extraction solvent. Throughout the procedure, the temperature was carefully maintained below 4 °C to preserve pigment stability. Chlorophyll a (Chl a), chlorophyll b (Chl b), and total carotenoid concentrations were estimated spectrophotometrically according to the correction equations described by Lichtenthaler and Buschmann [29], based on absorbance measurements at specific wavelengths, as follows:

$$Chl\;a\left({\mu g\;mL}^{-1}\right)=12.25\times A_{663.2}-2.79\times A_{646.8}$$

$$Chl\;b({\mu g\;mL}^{-1})=21.50\times A_{646.8}-5.10\times A_{663.2}$$

$$Total\;carotenoids\left({\mu g\;mL}^{-1}\right)=\left(1000\times A_{470}-1.82_{Cha}-85.02_{Chb}\right)/198$$

### 2.7. Statistical Analysis

In the present study, all experimental data from the biochemical analyses were subjected to one-way ANOVA using the Statistica 12 software package for Windows (StatSoft Inc., Tulsa, OK, USA), followed by Tukey’s multiple comparison test. Graphical representations were generated using SigmaPlot 12.0 (Systat Software).

## 3. Results and Discussion

### 3.1. Deciphering the Microbiome Profile of Hydroponic Nutrient Solution Before and After Photosynthetic Microorganism Bloom

To address the increasing demands of the growing human population, hydroponics is emerging as a promising alternative [5]. While hydroponics offers several advantages, it also presents challenges that must be overcome. One such challenge is the management of nutrient-rich hydroponic effluent, which remains a largely unexplored source of biodiversity with significant potential for bioprospecting. In addition, limited data on the composition of the respective microbial communities are currently available. Microbial communities can adapt to and thrive in such environments, with their composition influenced by factors such as cultivation practices and environmental conditions [30]. Due to the unique properties of hydroponic effluents, microalgae are among the microorganisms capable of efficiently utilizing the available inorganic nutrients through photosynthesis [11,31]. Thus, in the present study, we analyzed the microbial photosynthetic community present in the effluent of widely used tomato hydroponic systems, as well as its composition after the natural bloom of photosynthetic microorganisms.

Hydroponic effluent samples were collected after the end of the cultivation cycle without any other treatment, and their physicochemical properties were measured (Supplementary Table S1). Samples were then enriched with L1 medium and incubated under stable conditions until algal blooms were achieved. To decipher the microbiome profile before and after the algal bloom, we conducted a shotgun metagenomics analysis in duplicate samples (Supplementary Tables S3 and S4). Our goal was to estimate the relative abundance of photosynthetic microorganisms before the microbiological isolation process.

Two distinct shotgun metagenomic libraries were analyzed for each treatment. In total 93.856.404 single-end high-quality reads were analyzed before the algal bloom, and 71.918.136 single-end reads were analyzed after the algal bloom (Table 1). At first, a beta diversity analysis was conducted with a pairwise Bray–Curtis index of dissimilarity between the before and after algal bloom libraries individually, which showed, on average, a Bray–Curtis index of 0.651, revealing significant differences in the composition of the respective microbial communities. Upon the algal bloom, all alpha diversity indexes appeared significantly lowered, although without statistical significance. Shannon’s diversity showed an apparent reduction of approximately 2.5-fold (*p* = 0.353); Simpson’s index diversity appeared to have had a 2-fold decrease (*p* = 0.365), and whole Fisher’s index showed a 1.17-fold decrease (*p* = 0.767) (Table 2).

**Figure 1:**
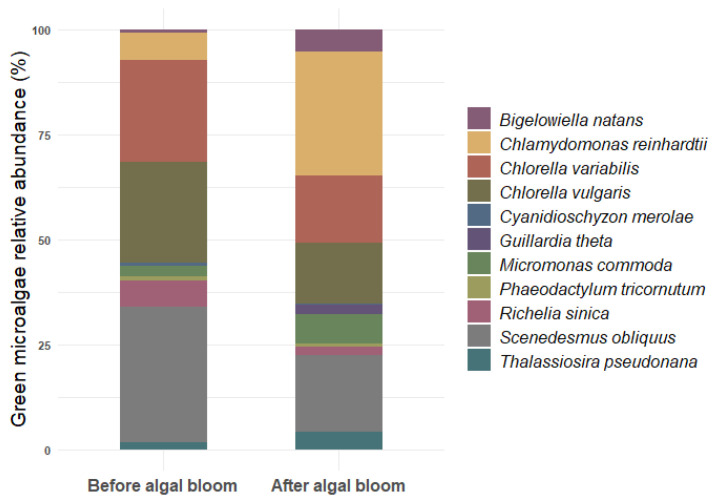
Average relative RPMs of photosynthetic microbes presented in this study at species level (using the Kraken 2 database and manual mapping) before and after algal bloom. One-hundred percent corresponds to the total RPMs of the strains studied (*n* = 2).

By utilizing two distinct methodologies, we were able to monitor a total of 11 photosynthetic microorganisms before and after the algal bloom (Figure 1). Initially, the utilization of the Kraken 2 database for microbial taxonomy resulted in the identification of 8 photosynthetic microorganisms (Supplementary Table S5). Our results revealed a 2.5-fold reduction in *Richelia sinica* (Cyanophyceae) RPMs (Reads Per Million), a 6-fold increase in RPMs of *Chlamydomonas reinhardtii* (Chlorophyceae), a 3-fold increase in *Micromonas commode* (Mamiellophyceae), a 4-fold increase in RPMs of *Thalassiosira pseudonana* (Coscinodiscophyceae), no difference in RPM of *Phaeodactylum tricornutum* (Bacillariophyceae), a 10-fold increase in *Bigelowiella natans* (Chlorarachniophyceae), and a significant enrichment of *Guillardia theta* (Cryptophyceae), for which no DNA fragments were detected before algal bloom, while *Cyanidioschyzon merolae* (Bangiophyceae) was at least 2-fold reduced after algal bloom. The evaluation of relative abundances of green photosynthetic microbes before and after the algal blooms allowed the identification of native microalgal species naturally occurring in the hydroponic effluent. Native microalgae already acclimated to the extreme conditions of wastewater environments can be identified, and may hold significant biotechnological potential. For instance, analysis of urban municipal wastewater and other waste sources revealed the presence of several microalgal species, including representatives of the genera *Chlorella*, *Tetradesmus*, and *Scenedesmus* [32]. Our results revealed the presence of a robust community of photosynthetic microorganisms compiled from at least eight species in relatively high abundance, each belonging to a distinct class.

This analysis confirmed the presence of *Chlorella vulgaris* (Trebouxiophyceae), *Chlorella variabilis* (Trebouxiophyceae), and *Scenedesmus rubescens* (Chlorophyceae) strains in the hydroponic effluent. Specifically, *C. vulgaris* and *C. variabilis* showed a 0.3-fold increase in RPM, although *C. vulgaris* was significantly more abundant, and Scenedesmus obliquus RPM increased 0.5-fold after the algal bloom. *Chlorella* species have been shown to thrive in hydroponic effluent, contributing to nutrient recycling by converting residual nutrients into microalgal biomass, thus supporting a wastewater-based circular bioeconomy [33]. Studies of effluents from cucumber and lettuce hydroponic systems revealed that *Chlamydomonas* spp. is a dominant representative of the algal community, although its effect on crops depends on various factors [31,34]. Taken together, a potential strategy for the bioremediation of hydroponic effluents and mitigation of their high nutrient content—a major obstacle to safe disposal into aquatic environments—could be the induction of natural algal blooms within the effluent. Indigenous microalgae could proliferate at minimal cost, consuming excess inorganic nutrients while simultaneously generating biomass. For instance, *Chlorella vulgaris* has been shown to grow cost-effectively in hydroponic water [35], and its presence has been associated with enhanced nitrogen and phosphorus removal [36]. Notably, as shown in the present study, the produced biomass includes species of high biotechnological value, including *Scenedesmus*, *Chlamydomonas* and *Chlorella* species, which can be exploited in downstream processes. This approach provides a dual benefit: improving effluent quality for environmental release with ‘zero residual nutrient discharge’ while producing valuable biomass at low cost.

In conclusion, the metagenomic shifts describe a transition from a complex wastewater microbiome to one dominated by phototroph strains belonging to known, cultivable genera like *Chlorella*, *Chlamydomonas* and *Scenedesmus*. This reduction in diversity and the rise of a phototrophic-controlled consortium indicate the system’s improved bioremediation capacity and create a stable foundation for biomass valorization, transforming the hydroponic effluent from a waste stream into a potent cultivation medium for high-value biomass. The identified native strains, already acclimated to the hydroponic effluent’s specific nutrient profile, represent prime candidates for scalable cultivation, using waste to produce a valuable biological resource while significantly reducing the nutrient load of the treated effluent. Thus, if released, it would exert far less environmental pressure on receiving ecosystems, mitigating the primary driver of eutrophication—harmful algal blooms, oxygen depletion, and biodiversity loss.

### 3.2. Microalgal Strains from Hydroponic Effluent Algal Bloom

**Figure 2:**
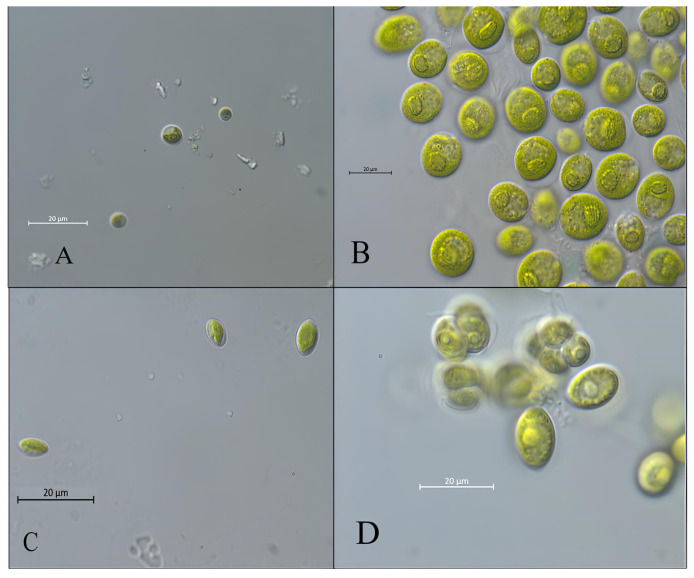
Light microscopy images of the four isolated microalgal strains. (**A**) *Chlorella* sp. strain PR1; (**B**) *Chlamydomonas* sp. (PR2); (**C**) *Chlorellaceae* (PR3); (**D**) *Scenedesmus rubescens* (PR4).

Starting from the algal blooms following the enrichment of hydroponic effluent samples, four distinct microalgal strains were successfully isolated and designated as PR1 to PR4. Morphological analysis has long served as a fundamental tool in the taxonomic identification of microalgae, particularly in the preliminary characterization of newly isolated strains. Features such as cell size, shape, presence or absence of flagella, chloroplast morphology, and colony formation offer valuable insights into the classification of microalgal taxa. However, morphological assessment alone is insufficient for microalgal taxonomy, and combining morphological, molecular, and ecological data provides a more robust framework for accurate and reliable classification [37]. Strain PR1 exhibited small, unicellular, spherical to subspherical cells with a diameter ranging between 4 and 8 μm (Figure 2). The cell wall was visibly rigid and smooth, without spines or ornamentation. Cells appeared mostly solitary, though under stress conditions (e.g., nutrient depletion), rare aggregates were observed. The chloroplast was parietal, occupying much of the intracellular space, with visible pyrenoids in most cells under standard light microscopy; all these traits are typical of the Chlorellaceae family [38]. Strain PR2 was easily distinguishable due to its motility and flagellar apparatus, with its traits being typical of the genus *Chlamydomonas* [39]. Cells were ovoid, measuring approximately 10–15 μm in diameter. Two anterior, equal-length flagella were consistently observed, conferring active motility. The cells possessed a large, cup-shaped chloroplast, within which one prominent pyrenoid was visible. A distinct eyespot (stigma) was present, located near the anterior region, confirming phototactic capacity. This strain often exhibited active rotation and directional swimming under light microscopy. Strain PR3 resembled members of the family Chlorellaceae, showing a more ellipsoidal and elongated morphology, with dimensions between 5 and 9 μm in length. The cell wall was flat and like PR1, cells rarely formed loose, irregular clusters. The chloroplast structure was pronounced, maintaining a parietal arrangement, with pyrenoids being occasionally visible. Finally, strain PR4 exhibited traits typical of the *Scenedesmus rubescens* species. Specifically, it presented larger, ellipsoidal cells with an average size of 6 × 15 μm. Cells typically occurred in unstructured clusters of two to four individuals, lacking the well-organized coenobial patterns typical of some classic *Scenedesmus* species [40]. The absence of flagella even during vegetative growth was a notable trait. Each cell contained a single, large chloroplast with a clearly visible pyrenoid, enclosed within a smooth, thin cell wall. The presence of longitudinal cell orientation and parallel alignment among some grouped cells suggested an incomplete tendency toward colonial structure, typical of this genus. Moreover, under extreme stress conditions, notable alterations in both morphology and pigment composition were exhibited. Cells developed thicker walls, chloroplasts became more condensed, and enhanced accumulation of secondary carotenoids was observed within the plastids, imparting a reddish or orange hue to the cells, all traits typical of *S. rubescens* [41].

To elucidate the phylogenetic relationships of the isolated microalgal strains, a DNA barcoding approach was employed using four widely accepted molecular markers: the nuclear 18S rRNA gene, the internal transcribed spacer (ITS) region, and the plastid *rbcL* and *tufA* genes [42,43,44,45,46,47]. Partial sequences of all markers were obtained from the isolates, and subsequently aligned and trimmed to uniform lengths. Phylogenetic trees were then generated individually, employing both Neighbor-Joining (NJ) and Maximum Likelihood (ML) methods to infer evolutionary relationships. Tree topologies were largely congruent across markers and methods, with only minor variations in the placement of taxa within clades. For all markers, ML trees, which yielded higher bootstrap support values, were selected for interpretation (Supplemental Figures S1–S4).

The nuclear markers 18S rDNA and ITS consistently delineated the isolates into distinct, well-supported lineages. The 18S rDNA phylogeny resolved the microalgal strains into three robust clades (Supplemental Figure S1). The PR1 isolate clustered within the *Micractinium–Chlorella* lineage, exhibiting up to 99.94% pairwise sequence identity with *Micractinium inermum* NIES:2171 strain and 99.82% with other related taxa. PR3 could not be included in the full 18S phylogeny due to incomplete sequence data. However, BLAST analysis of a 576 bp fragment supported its placement within the Chlorellaceae family, showing high sequence identity (99.26%) to *Meyerella planktonica* strains. The PR4 isolate grouped within the Scenedesmaceae family, sharing 99.94–100% identity with related genera such as *Scenedesmus*, *Tetradesmus*, *Scotiellopsis*, *Acutodesmus* and *Coelastrum*. Finally, PR2 grouped with *Chlamydomonas*, exhibiting 100% identity with *C. uva-maris* SAG 19.89 and 98.63–98.69% similarity to other *Chlamydomonas* strains. The ITS region further corroborated these relationships while providing greater resolution (Supplemental Figure S2). PR1 again clustered with *Micractinium* and *Chlorella*, exhibiting 99.54% pairwise sequence similarity. Interestingly, despite their close resemblance in the 18S phylogeny, PR3 and PR4 shared only 35.20% ITS similarity, highlighting the greater discriminatory power of the ITS marker. Specifically, PR3 formed a distinct lineage affiliated with its closest relative, *Meyerella planktonica*, sharing only 88.33% sequence similarity. In contrast, PR4 showed strong sequence similarity (99.85–100%) with *Scenedesmus, Tetradesmus*, and *Scotiellopsis* strains. PR2 could not be resolved in the ITS phylogeny due to incomplete sequence data. However, BLAST analysis of a 368 bp fragment supported its affiliation with *Chlamydomonas*, although with relatively low sequence identity (92.92%) to *C. uva-maris* strains.

Plastid markers (*rbcL* and *tufA*) provided complementary resolution and consistently delineated the isolates into four well-supported clades (I–IV). In the *rbcL* phylogeny (Supplemental Figure S3), PR1 clustered within the *Chlorella–Micractinium* group, exhibiting 97.36–98.45% pairwise sequence similarity to reference strains in this lineage. PR3 formed a distinct branch with *Chlorella* sp. M9, although its sequence similarity with other Chlorellaceae reference taxa in the clade was relatively low (90.85–92.87%). PR4 grouped within the Scenedesmaceae clade, displaying 100% identity with *Tetradesmus* sp. NAMSU 221a and *Halochlorella rubescens* KNUA042, and 98.91–99.22% sequence similarity with other related strains in the clade. Isolate PR2 was positioned within the *Chlamydomonas* group, showing 89.77% sequence similarity to *Chlamydomonas* sp. ICE-L. The *tufA* phylogeny (Supplemental Figure S4) was largely congruent with the *rbcL* tree, with only minor discrepancies within the clades. PR1 grouped with *Chlorella* sp. ArM0029B (98.24%), *Micractinium simplicissimum* (97.88%) and *Micractinium singularis* MM0003 (97.77%), and it exhibited lower similarity (89.19–93.18%) to other *Chlorella–Micractinium* strains. PR3 clustered with *Marvania geminata* SAG 12.88, sharing only 84.72% sequence similarity and remaining distantly related to other taxa in the clade (83.08–86.02% sequence similarity). PR2 was resolved within the *Chlamydomonas* lineage, displaying 90.66% sequence similarity to *Chlamydomonas* sp. ICE-L. The PR4 isolate was consistently positioned within the *Scenedesmus–Tetradesmus* clade, sharing 97.61–99.54% sequence similarity with related taxa.

**Figure 3:**
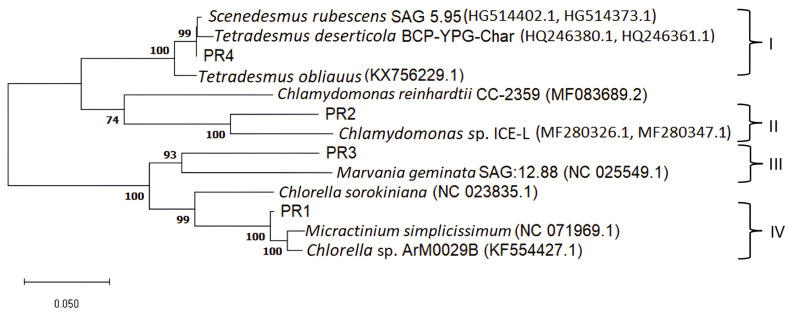
Phylogenetic ML tree inferred under the best-fit substitution model (GTR+G), based on *rbcL* and *tufA* concatenated gene sequences, showing the relationships of microalgal isolates with their related species retrieved from GenBank database. The evolutionary rate differences among sites were modeled using a discrete Gamma distribution across 5 categories (+G, parameter = 0.3893). Bootstrap values (calculated for 1000 replicates) > 70% are shown on the branches. Scale bar = 0.050% substitutions per site.

Combining the plastid markers into a concatenated phylogenetic tree further enhanced resolution, clearly delineating the isolates into the same four clades (I–IV) (Figure 3). PR1 grouped within the Chlorellaceae family, sharing 97.79–97.93% sequence identity with reference taxa, and was most closely related to *Chlorella* sp. ArM0029B, a strain isolated from an Arctic environment [48]. PR2 clustered with *Chlamydomonas* species, showing 90.28% similarity to *Chlamydomonas* sp. ICE-L, which was originally isolated from Antarctic ice and classified based on both morphological and molecular characteristics [49]. PR3 was placed within the Chlorellaceae family, exhibiting 87.43% identity with *Marvania geminata* SAG 12.88, a strain previously assigned to the Chlorellales group using a chloroplast phylogenomic approach [50]. PR4 showed 99.41% identity with *Scenedesmus rubescens* SAG 5.95. Although this strain was formerly classified within the *Chlorella* genus, it is now considered part of the *Scenedesmus* genus [51,52].

Overall, the multilocus phylogenetic analyses confirm that the four isolated strains represent distinct lineages within green algal families: (i) PR1 belongs to the Chlorellaceae family, specifically the *Chlorella–Micractinium* group (*Chlorella* sp.); (ii) PR3 also falls within the Chlorellaceae family but forms a distinct lineage related to *Meyerella* and *Marvania* genera; (iii) PR2 is affiliated with the Chlamydomonadaceae family and corresponds to *Chlamydomonas* sp.; and (iv) PR4 is positioned within the Scenedesmaceae family, corresponding to *Scenedesmus rubescens*. Although the 18S rDNA sequences indicated relatively high similarity among the isolates (92.38–94.29%), the higher-resolution markers revealed substantial genetic divergence, complicating accurate classification. These discrepancies may also reflect the limited availability of closely related sequences in existing reference databases. Pairwise sequence similarities declined markedly in the plastid genes (*rbcL*: 85.43–92.25%; *tufA*: 73.92–84.61%) and were particularly low in the ITS region (35.20–74.96%), highlighting the importance of using markers with varying evolutionary rates to resolve closely related taxa. PR3 clustered near the *Meyerella* and *Marvania* species, with low sequence similarity, yet exhibited substantial divergence from other Chlorellaceae taxa, including members of the *Chlorella–Micractinium* group. This pattern is consistent with current phylogenetic evidence indicating that, although *Meyerella* and *Chlorella* both belong to the Chlorellaceae family, they represent divergent evolutionary lineages. The low pairwise sequence similarity, the placement of PR3 in independent lineages within the chloroplast phylogenies, and the absence of additional distinguishing morphological traits suggest that this isolate warrants further investigation. Applying additional molecular markers will be necessary to better characterize and accurately classify it. Together, these findings confirm that all four isolates correspond to genetically and phylogenetically distinct taxa, demonstrating the value of integrative, multilocus approaches for robust microalgal identification and classification.

### 3.3. Growth Evaluation and Kinetics Measurements Under Different Temperature Regimes

**Figure 4:**
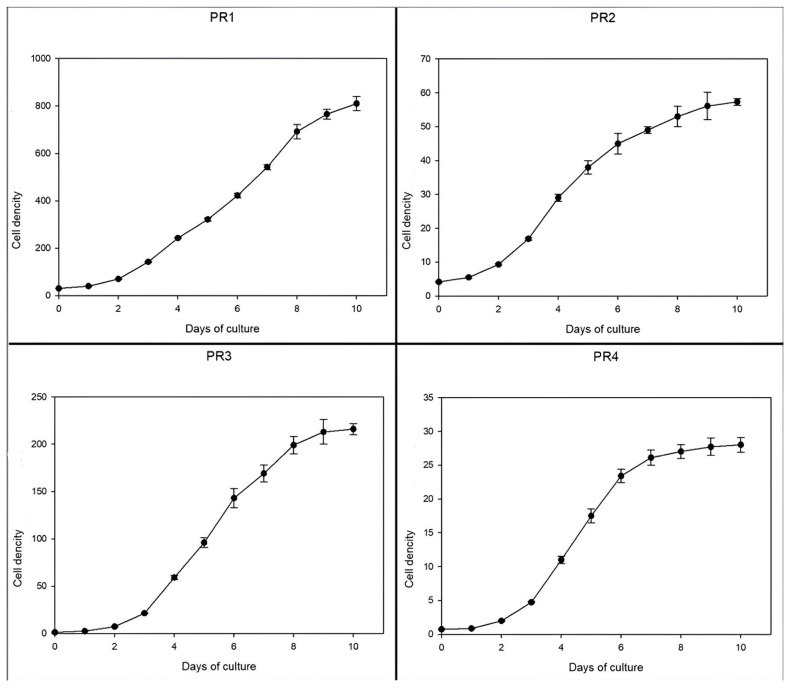
Growth curves of the isolated microalgal strains cultivated for 10 days in L1 medium under control conditions. Mean ± SD of three biological replicates. The cell density is expressed in cells/mL × 10^5^.

All four isolated microalgal strains demonstrated robust growth under control conditions (Figure 4), highlighting their potential for scalable cultivation. Notably, the *Chlorella* sp. strain PR1 exhibited rapid acclimation, entering the exponential growth phase by day 2 with a maximum specific growth rate (μ_max_) of 0.601 d^−1^ and a final cell density of 81 × 10^6^ cells mL^−1^. This performance aligns closely with the *Chlorella vulgaris* strain CCAP 211/11B [53], with a short lag phase and high productivity being characteristic traits of this genus. Similarly, the *Chlamydomonas* sp. strain PR2 achieved a μ_max_ of 0.554 d^−1^ and a cell density of 5.73 × 10^6^ cells mL^−1^, comparable to growth rates reported for other *Chlamydomonas* strains under analogous conditions [54]. In contrast, strain PR3 displayed near-instantaneous acclimation, bypassing a detectable lag phase entirely, and achieved the highest μ_max_ (1.054 d^−1^) among the isolates. By day 10, PR3 reached a cell density of 21.6 × 10^6^ cells mL^−1^, underscoring its exceptional biomass accumulation potential. The most striking performance, however, was observed in *Scenedesmus rubescens* strain PR4, which attained a μ_max_ of 0.846 d^−1^ and a cell density of 2.8 × 10^6^ cells mL^−1^. This represents a 40% higher growth rate compared to the *S. rubescens* strains reported by Lv et al. [55], potentially attributable to PR4’s adaptation to controlled laboratory conditions or unique physiological traits.

Collectively, these results suggest that the isolated strains exhibit growth kinetics that not only match but, in some cases, surpass established benchmarks for their genera. The absence of prolonged lag phases implies efficient light/nutrient utilization, a critical advantage for industrial bioreactors requiring rapid biomass turnover, while *Scenedesmus rubescens* strain PR4’s superior performance relative to the literature values further emphasizes the importance of strain-specific selection for optimizing cultivation protocols [55].

**Figure 5:**
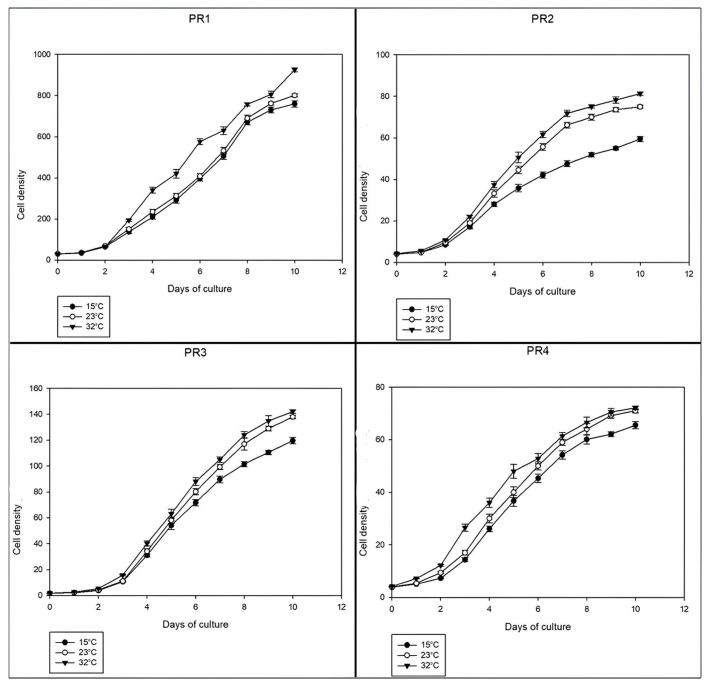
Growth curves of the isolated microalgal strains cultivated for 10 days in L1 medium under three different temperature regimes (15 °C, 23 °C and 32 °C). Mean ± SD of three biological replicates. The cell density is expressed in cells/mL × 10^5^.

Temperature plays a crucial role in microalgal growth, influencing both cell density and growth rates in natural environments and outdoor photobioreactors [56]. To evaluate the adaptability and further support the biotechnological potential of the isolated strains, they were cultivated under different temperature conditions commonly used in microalgal research. Overall, all strains exhibited successful growth across the tested temperature range, with cell densities and growth rates generally increasing at higher temperatures (Figure 5).

Among the strains analyzed, *Scenedesmus rubescens* strain PR4 exhibited the most pronounced response to temperature variations. Growth at 32 °C resulted in a more than 40% increase in final cell density and a 22% increase in maximum growth rate compared to growth at 15 °C. This suggests that this strain may have a broad thermal tolerance, making it a strong candidate for large-scale cultivation in varying environmental conditions, suitable for the Mediterranean region. *Chlorella* sp. strain PR1 also showed a notable response to temperature, with cultivation at 32 °C leading to a 22% increase in final cell density and a 25% enhancement in maximum growth rate relative to 15 °C. These results are consistent with studies on *Chlorella vulgaris* strains, which exhibited optimal biomass concentration and productivity at 25 °C following successful cultivation at 15 °C, 25 °C, and 35 °C [57].

For *Chlamydomonas* sp. strain PR2, temperature had a different effect. Although its maximum specific growth rate remained relatively unchanged between 15 °C and 32 °C, its final cell density increased by 37%. This increase can be attributed to the slower decline in growth rate after the exponential phase, which was observed both at 23 °C and 32 °C. This suggests that this strain maintains sustained growth over extended periods at higher temperatures, a characteristic that may be advantageous for biomass production. Among the four strains, PR3 was the least affected by temperature changes. Its maximum growth rate remained largely unchanged, and its final cell density showed the smallest increase (19%). This stability suggests that PR3 is well-adapted to a broad range of temperatures, maintaining consistent growth performance regardless of moderate fluctuations.

While some species, such as *Chaetoceros* sp., *Tetraselmis suecica*, and *Nannochloropsis* sp., have been shown to exhibit decreased specific growth rates and biomass content at higher temperatures [56], the strains in this study demonstrated considerable adaptability, showcasing their ability to grow efficiently across multiple temperature regimes and underscoring their potential for mass cultivation under diverse environmental conditions.

### 3.4. Biochemical Profiling and Valorization of Microalgal Biomass

**Figure 6:**
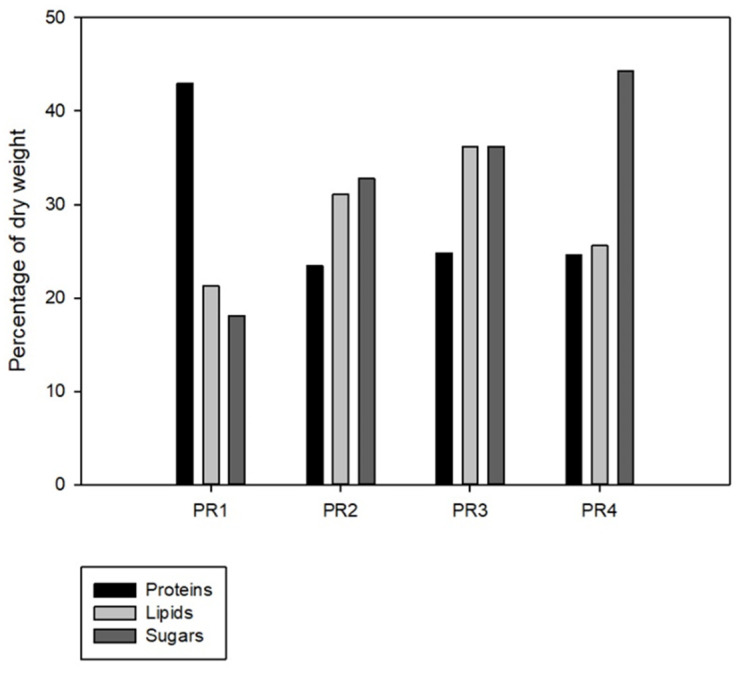
Content of proteins, lipids and sugars in the isolated strains expressed as percentage of biomass dry weight.

The biochemical profiles of the isolated strains, including major cell components (proteins, lipids, sugars), photosynthetic pigments (chlorophyll and carotenoids), secondary metabolites (phenolics and flavonoids), and antioxidant activity, revealed significant interspecific variability (Figure 6; Table 3). *Chlorella* sp. strain PR1 emerged as a protein-rich candidate (43% *w*/*w* DW), significantly exceeding all other isolated strains, though slightly below the upper range reported for *Chlorella* sp. [24,58]. In contrast, *Chlamydomonas* sp. strain PR2, PR3, and *Scenedesmus rubescens* strain PR4 prioritized lipid (21–39% *w*/*w* DW) and sugar (18–44% *w*/*w* DW) accumulation, showcasing considerably lower protein contents than those reported for *Chlamydomonas* and *Scenedesmus* strains (48 and 50–56%, respectively) [58]. It is noteworthy that *S. rubescens* strain PR4’s sugar content (44% *w*/*w* DW) was the highest among the isolated strains, exceeding typical values for *Scenedesmus* species (e.g., 30.5% *w*/*w* DW in Visca et al., [59]), while *Chlamydomonas* sp. strain PR2 exhibited a lipid content (31% *w*/*w* DW) almost two times greater than that reported by Metsoviti et al. [60] for a *Chlamydomonas reinhardtii* strain (15.8% *w*/*w* DW). This divergence likely reflects the strains’ metabolic adaptation to nitrogen/phosphorus-poorer L1 medium, given their origin from nutrient-rich hydroponic effluent, with limited nutrient availability being one of the most important factors affecting microalgae biomass composition [61]. Many researchers have shown how phosphorus and, more importantly, nitrogen depletion in the culture medium could trigger a metabolic shift from protein to lipid and sugar synthesis [62].

The pigment profile of the isolated microalgal strains varied significantly, with *Chlorella* sp. strain PR1 exhibiting the highest chlorophyll content (14.68 mg/g DW of chlorophyll a and 8.19 mg/g DW of chlorophyll b) surpassing many *Chlorella* strains, whereas *S. rubescens* strain PR4 displayed the lowest levels (1.75 mg/g DW of chlorophyll a and 1.27 mg/g DW of chlorophyll b), with all four strains displaying values aligning with literature-reported ranges for *Chlorella*, *Scenedesmus* and *Chlamydomonas* species [63,64,65,66]. Carotenoid concentrations revealed a slightly different result, with *Chlamydomonas* sp. strain PR2 and strain PR3 displaying the highest total carotenoid contents (3.06 and 3.00 mg/g DW, respectively), followed by *Chlorella* sp. strain PR1 (1.86 mg/g DW), while *Scenedesmus* sp. strain PR4 again recorded the lowest concentration (0.54 mg/g DW). These findings align with genus-specific trends, as *Chlorella* strains consistently yielded carotenoid levels within the range reported in the literature (2–4 mg/g DW) depending on the specific strain and cultivation conditions [63,67,68], with the same being true for *Chlamydomonas* and *Scenedesmus* strains [64,69].

Flavonoids and phenolic compounds are pivotal secondary metabolites renowned for their antioxidant and stress-ameliorating properties, with the interspecific variability in their accumulation not only underscoring genus-specific metabolic strategies but also highlighting the potential for valorizing these bioactive compounds in various applications. *Chlamydomonas* sp. strain PR2 and PR3 emerged as standout strains for phenolic and flavonoid production, accumulating 4.48 and 3.99 mg gallic acid equivalents (GAE)/g dry weight (DW) of total phenolics and 3.57 and 3.56 mg catechin equivalents (CE)/g DW of flavonoids, respectively. Both strains exhibited phenolic contents significantly higher than those reported for *Chlorella* (0.75 to 3.69 mg GAE/g DW) and *Chlamydomonas* (2.4 to 2.5 mg GAE/g DW) species [60,65,70,71]. While strain PR3 also exhibited higher flavonoid content compared to the reported *Chlorella* species (around 2.4 mg QE/g DW), *Chlamydomonas* sp. strain PR2 exhibited slightly lower levels than those reported by Grande et al. [72] for *Chlamydomonas agloeformis* (4.4 mg QE/g DW). *Chlorella* sp. PR1 exhibited moderate phenolic (2.95 mg GAE/g DW) and flavonoid levels (1.38 mg QE/g DW) that were in sync with those reported in the literature for strains belonging to the *Chlorella* genus, while the *S. rubescens* strain PR4 exhibited the lowest levels among the isolated strains for both phenolic (0.88 mg GAE/g DW) and flavonoid (1.08 mg QE/g DW) content. These values were not only lower than those exhibited by the isolated strains but also lower than the phenolic (1.94 mg GAE/g DW) and flavonoid content (1.61 mg QE/g DW) reported for the *Scenedesmus* genus by Goiris et al. [73] and Bulut et al. [74], respectively.

Antioxidant activity in microalgae serves as a critical physiological indicator of their capacity to mitigate oxidative stress induced by environmental perturbations, with the quantification of this activity reflecting not only strain-specific adaptive strategies but also underscoring their potential as sustainable sources of natural antioxidants for industrial applications. The antioxidant activity of the isolated microalgal strains varied significantly, with strain PR3 and *Chlamydomonas* sp. strain PR2 exhibiting the highest antioxidant capacity, with values of 13.86 and 18.86 mg Trolox/g DW, respectively, followed by *Chlorella* sp. strain PR1, which recorded 6.29 mg Trolox/g DW, and *S. rubescens* strain PR4 demonstrated the lowest antioxidant activity, measuring 4.31 mg Trolox/g DW. It is noteworthy that these results correlate strongly with the measured carotenoid, phenolic and flavonoid content, underscoring the well-established role of these metabolites as primary contributors to antioxidant defense in microalgae [75]. The antioxidant activity of the isolated strains was higher than that reported for similar strains in the literature, with various strains of *Chlorella vulgaris* exhibiting antioxidant activity values ranging from 1.35 to 4.99 mg Trolox/g DW, significantly lower than the values measured for *Chlorella* sp. strain PR1 and PR3 [65,70]. Similarly, Vieira et al. [65] reported a value of 6.03 mg Trolox/g dry biomass for a strain of *Chlamydomonas nivalis*, which was lower than the value observed in strain PR2. It is highly interesting that strain PR4 exhibited an antioxidant activity value higher than that reported by Goiris et al. [73] for a *Scenedesmus obliquus* strain (1.47 mg Trolox/g dry biomass). This result combined with the relatively low values of phenolic, flavonoid and carotenoid levels suggests that this strain may use other antioxidant mechanisms and accumulate different antioxidant metabolites. Finally, it is important to note that antioxidant activity can vary significantly among strains due to genetic differences, but it is also heavily influenced by the physiological state of the microalgae. Since the strains used in this study were cultured in a medium with lower nitrogen and phosphorus levels than the environment from which they were isolated, they may be subjected to environmental stress, which has been shown to increase antioxidant activity in microalgae [75].

All isolated strains demonstrated remarkable biochemical prowess, rivaling or surpassing well-characterized representatives of their respective genera. *Chlamydomonas* sp. strain PR2 and PR3 outperformed *C. reinhardtii* lipid benchmarks by >15% and eclipsed *Chlorella* sp. phenolic/flavonoid norms by 20–35%, while *Chlorella* sp. strain PR1 maintained protein levels comparable to industrial *Chlorella* strains despite nutrient deficiency. Even *S. rubescens* strain PR4 exceeded typical *Scenedesmus* carbohydrate yields by 44%, defying genus-specific metabolic expectations. Such exceptional performance underscores these strains’ adaptive resilience and positions them as high-potential candidates for tailored applications—bridging ecological niche adaptation with biotechnological innovation.

The demonstrated biochemical diversity and superior metabolite accumulation among these newly isolated strains highlight their multifaceted potential for high-value product development. Elevated lipid and carbohydrate profiles position *Chlamydomonas* sp. PR2, PR3 and *S. rubescens* PR4 as promising feedstocks for sustainable biofuel and biopolymer production, while the pronounced phenolic and flavonoid contents in *Chlorella* sp. PR1 strain coupled with the robust protein retention observed under nutrient limitation underscore applications in antioxidant-rich nutraceuticals, cosmeceuticals, and functional food formulations. Collectively, these findings emphasize the strategic importance of strain-specific valorization—linking ecological adaptability with scalable biotechnological exploitation—and pave the way for integrative biorefinery approaches targeting both energy and health-oriented markets. 

## 4. Conclusions

Currently, the microbial communities thriving in hydroponic effluents are largely unexplored, although they hold promise for the isolation of microbes with bioprospecting potential. In this study, we examined the photosynthetic microbial consortium found in hydroponic effluent, with an emphasis on microalgal populations with putative biotechnological interest. Our study was limited by the low number of metagenomic replicates and the non-fixed timing of algal bloom community sampling. Consequently, limited conclusions can be drawn regarding shifts in microbial abundances from an ecological perspective. From a biotechnological point of view, however, the results clearly demonstrate that effluent water after an algal bloom can be successfully exploited for the isolation of valuable photosynthetic microbes, highlighting an understudied microbial reservoir. Specifically, representative strains were isolated, identified, and characterized at both kinetic and biochemical levels. A combination of morphological observations and multilocus phylogenetic analysis confidently assigned the isolated strains. Our isolates demonstrated exceptional growth performance and thermal adaptability, rivaling or even surpassing well-established representatives of their genera. Their high resilience, coupled with minimal lag phases, positions these strains as ideal candidates for scalable bioreactor systems, where consistent productivity under variable conditions is essential. They also exhibited promising biochemical properties, including enhanced antioxidant capacity. Future smartly designed biotechnological strategies could provide proof-of-concept for their utilization as biostimulants, nutraceuticals and pharmaceuticals.

## Figures and Tables

**Table 1:** Number of raw reads analyzed and their respective classification after Kraken 2 analysis.

	Number of Raw Reads	Classified Reads	Chordate Reads	Unclassified Reads	Microbial Reads	Bacterial Reads	Viral Reads
Before Algal Bloom	29,987,991	3,128,798	70,138	26,859,193	3,055,247	3,024,768	419
	16,872,682	5,246,396	25,980	11,626,286	5,219,118	5,200,169	9111
After Algal Bloom	13,914,993	11,353,400	22,075	2,561,593	11,331,060	11,327,903	118
	21,480,190	8,168,392	48,739	13,311,798	8,117,462	8,100,898	364

**Table 2:** Alpha and beta diversity indexes per treatment.

	Pairwise Average Bray–Curtis Index	Average Shannon’s Diversity	SE±	Average Simpson’s Index of Diversity	SE±	Average Fisher’s Index	SE±
Before Algal Bloom	0.651	3.30	0.90	0.73	0.17	138.42	9.61
After Algal Bloom		1.39	0.58	0.36	0.14	117.61	38.32

**Table 3:** Pigments, flavonoid and phenolic contents of isolated strains and their respective antioxidant activity (TEAC). Data are presented as mean values (*n* = 5) ± standard error (SE). Different letters indicate statistically significant differences among the microalgal strains (*p* < 0.05).

Strain	Chl a (mg/g DW)	Chl b (mg/g DW)	Carotenoids (mg/g DW)	Flavonoids (mg Catechin/g DW)	Phenolics (mg Gallic Acid/g DW)	TEAC (mg Trolox/g DW)
PR1	14.68 ± 0.313 ^a^	8.19 ± 0.343 ^a^	2.06 ± 0.068 ^b^	1.38 ± 0.111 ^b^	2.95 ± 0.242 ^b^	6.62 ± 0.083 ^c^
PR2	10.54 ± 0.221 ^b^	5.63 ± 0.154 ^b^	3.06 ± 0.022 ^a^	3.57 ± 0.062 ^a^	3.99 ± 0.059 ^a^	13.86 ± 0.380 ^b^
PR3	8.62 ± 0.131 ^c^	5.26 ± 0.095 ^b^	3.00 ± 0.043 ^a^	3.56 ± 0.074 ^a^	4.48 ± 0.058 ^a^	19.08 ± 0.139 ^a^
PR4	1.75 ± 0.015 ^d^	1.27 ± 0.009 ^a^	0.54 ± 0.012 ^c^	1.08 ± 0.026 ^b^	0.88 ± 0.041 ^c^	4.31 ± 0.668 ^d^

## Data Availability

All the raw sequence files generated from the metabolomic analysis of this study were submitted to the European Nucleotide Archive (ENA) [23] with the study accession number PRJEB82461 and are available at http://www.ebi.ac.uk/ena/data/view/PRJEB82461, accessed on 20 February 2026. Sequence information generated for the molecular taxonomy is deposited in the NCBI database with the accession numbers PQ773481-PQ773488 abd PQ800404-PQ800407. All data are available upon request to the authors.

## Supplementary Materials

The following supporting information can be downloaded at: https://www.mdpi.com/article/10.3390/microorganisms14030582/s1, Supplementary Figure S1: Phylogenetic ML tree inferred under the best-fit substitution model (K2+I), based on 18S rDNA region (1672 nt), showing the relationships of microalgal isolates with closely related species obtained from GenBank database. The rate model allowed for 81.68% of sites to be evolutionarily invariable (I). Bootstrap values (calculated for 1000 replicates) > 70% are shown on the branches. Scale bar = 0.01% substitutions per site; Supplementary Figure S2: Phylogenetic ML tree inferred under the best-fit substitution model (K2), based on ITS region (616 nt), showing the relationships of microalgal isolates with closely related species obtained from GenBank database. Bootstrap values (calculated for 1000 replicates) > 70% are shown on the branches. Scale bar = 0.1% substitutions per site; Supplementary Figure S3: Phylogenetic ML tree inferred under the best-fit substitution model (GTR+G), based on rbcL gene sequences, showing the relationships of microalgal isolates with their related species retrieved from GenBank database. The evolutionary rate differences among sites were modeled using a discrete Gamma distribution across 5 categories (+G, parameter = 0.2555). Bootstrap values (calculated for 1000 replicates) > 70% are shown on the branches. Scale bar = 0.050% substitutions per site; Supplementary Figure S4: Phylogenetic ML tree inferred under the best-fit substitution model (GTR+G+I), based on tufA gene sequences, showing the relationships of microalgal isolates with their related species retrieved from GenBank database. The evolutionary rate differences among sites were modeled using a discrete Gamma distribution across 5 categories (+G, parameter = 1.2721), with 41.59% of sites deemed evolutionarily invariant (+I). Bootstrap values (calculated for 1000 replicates) >70% are shown on the branches. Scale bar = 0.050% substitutions per site; Supplementary Table S1: Physicochemical properties of HE; Supplementary Table S2: Primers sequences used for molecular taxonomy; Supplementary Table S3: Taxonomic groups identified using Bracken in both replicates of hydroponic effluent samples before algal bloom; Supplementary Table S4: Taxonomic groups identified using Bracken in both replicates of hydroponic effluent samples after algal bloom; Supplementary Table S5: Normalized reads per million (RPM) before and after algal bloom of photosynthetic microbes after Kraken 2 taxonomy. Bold microorganisms correspond to whole genome mapping of the genomic libraries.
