# Supplementary material for: Isolation and Characterization of Microalgae Isolates from Hydroponic Effluent Water: Metagenomics and Biotechnological Insights
Source: Microorganisms. 2026 Mar 4;14(3):582. doi: 10.3390/microorganisms14030582 (PMC13029762; doi:10.3390/microorganisms14030582)

Supplementary Figure S1. Phylogenetic ML tree inferred under the best-fit substitution model (K2+I), based on 18S rDNA region (1672 nt), showing the relationships of microalgal isolates with closely related species obtained from GenBank database. The rate model allowed for 81.68% of sites to be evolutionarily invariable (*I*). Bootstrap values (calculated for 1000 replicates) >70% are shown on the branches. Scale bar = 0.01% substitutions per site.

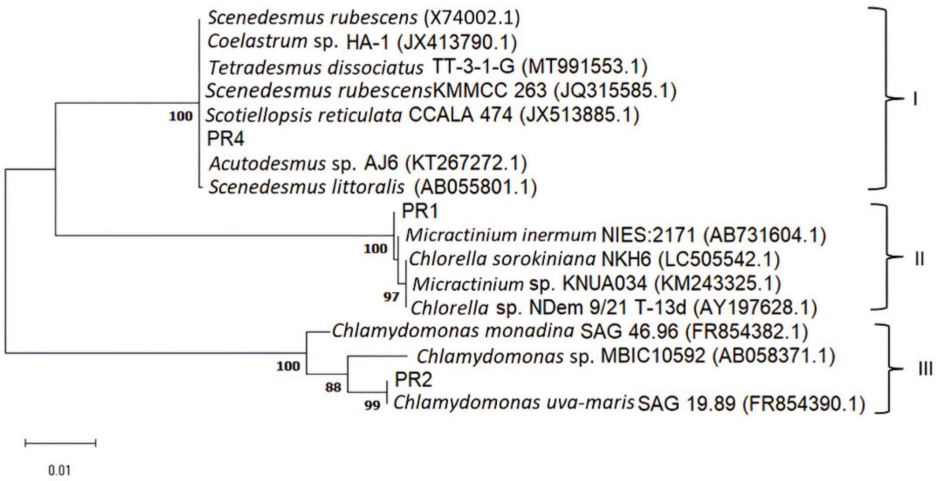

Supplementary Figure S2. Phylogenetic ML tree inferred under the best-fit substitution model (K2), based on ITS region (616 nt), showing the relationships of microalgal isolates with closely related species obtained from GenBank database. Bootstrap values (calculated for 1000 replicates) >70% are shown on the branches. Scale bar = 0.1% substitutions per site.

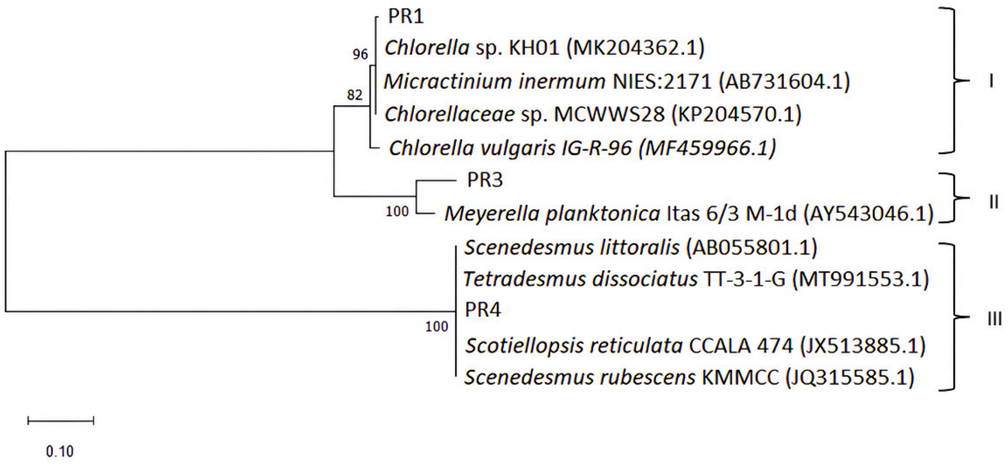

Supplementary Figure S3. Phylogenetic ML tree inferred under the best-fit substitution model (GTR+G), based on *rbcL* gene sequences, showing the relationships

of microalgal isolates with their related species retrieved from GenBank database. The evolutionary rate differences among sites were modeled using a discrete Gamma distribution across 5 categories (+*G*, parameter = 0.2555). Bootstrap values (calculated for 1000 replicates) >70% are shown on the branches. Scale bar = 0.050% substitutions per site.

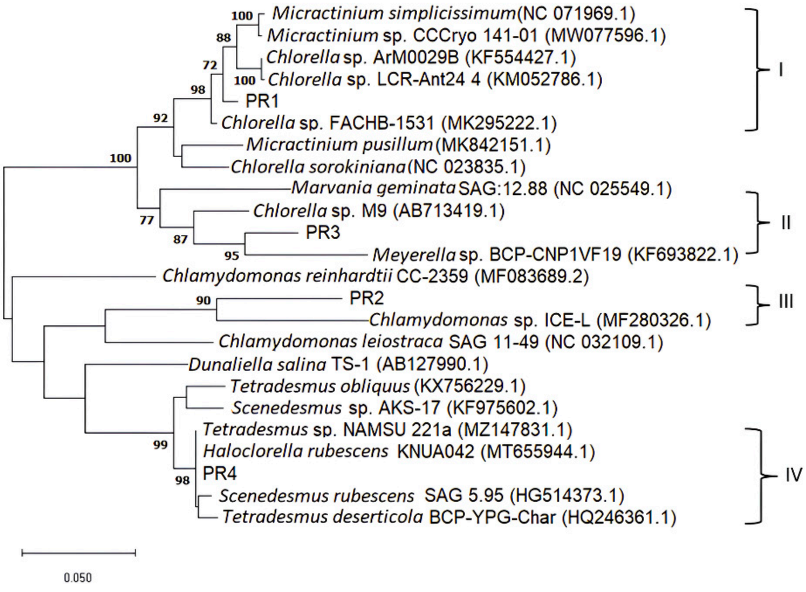

Supplementary Figure S4. Phylogenetic ML tree inferred under the best-fit substitution model (GTR+G+I), based on *tufA* gene sequences, showing the relationships of microalgal isolates with their related species retrieved from GenBank database. The evolutionary rate differences among sites were modeled using a discrete Gamma distribution across 5 categories (+*G*, parameter = 1.2721), with 41.59% of sites deemed evolutionarily invariant (+*I*). Bootstrap values (calculated for 1000 replicates) >70% are shown on the branches. Scale bar = 0.050% substitutions per site.

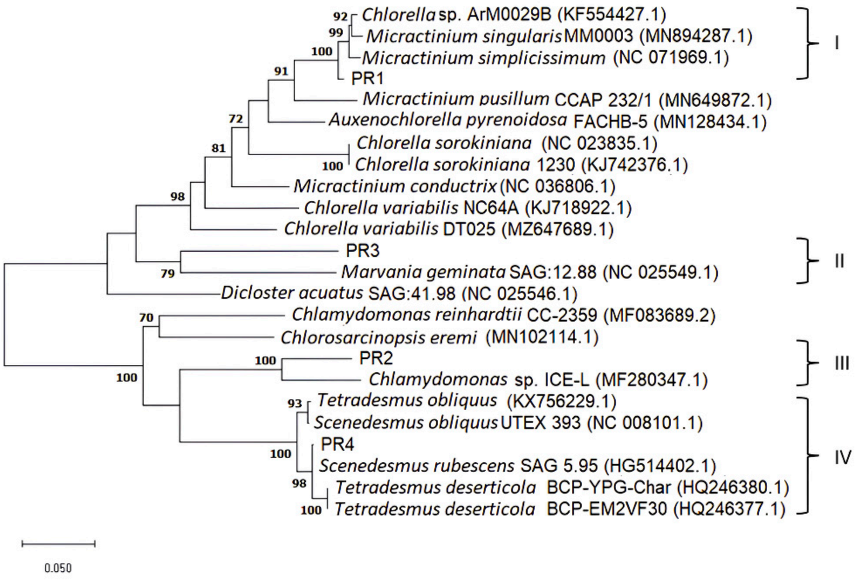

Supplement: Supplementary file 1 [file microorganisms-14-00582-s001.zip › Supplementary figures.pdf]
